# Effectiveness and implementation of an inpatient mental health care pathway at an epilepsy center: A prospective service evaluation

**DOI:** 10.1111/epi.70014

**Published:** 2025-11-14

**Authors:** Rosa Michaelis, Dorothea Hölscher, Katharina Braun, Sabine Schlömer, Wenke Grönheit, Tim Wehner, Claudia Grunert, Markus Reuber, Johannes Jungilligens, Jörg Wellmer, Friedrich Edelhäuser, Corinna Seliger, Stoyan Popkirov

**Affiliations:** ^1^ Ruhr Epileptology, Department of Neurology University Hospital Knappschaft Kliniken Bochum, Ruhr University Bochum Bochum Germany; ^2^ Faculty of Health University Witten/Herdecke Witten Germany; ^3^ Department of Neurology and Center for Translational and Behavioral Neurosciences (C‐TNBS) University Medicine Essen, University of Duisburg‐Essen Essen Germany; ^4^ Therapienetz Essstörung Munich Germany; ^5^ Forschungs‐ und Behandlungszentrum für Psychische Gesundheit Bochum Germany; ^6^ Department of Neurology University Hospital Knappschaft Kliniken Bochum, Ruhr University Bochum Bochum Germany; ^7^ Academic Neurology Unit University of Sheffield, Royal Hallamshire Hospital Sheffield UK

**Keywords:** antidepressants, anxiety, comorbidities, depression, health‐related quality of life, psychotherapy, seizure frequency

## Abstract

**Objectives:**

This hybrid study assessed the implementation and clinical effectiveness of a structured mental health care workflow for epilepsy.

**Methods:**

Eligible inpatients were screened systematically. Patients with scores above cutoff scores underwent structured diagnostic interviews followed by a multi‐component psychotherapeutic intervention (one or two sessions) aiming to develop a personalized treatment plan. Follow‐up at 1, 3, 6, and 12 months assessed treatment plan adherence and reliable change indices (RCIs) of outcomes (self‐reported depressive and anxiety symptoms, health‐related quality of life, work and social adjustment). Implementation was assessed through initial step penetration, fidelity of workflow execution, and diagnostic/therapeutic yields (appropriateness).

**Results:**

Of 345 inpatients with epilepsy, 210 were eligible and 202 entered screening. Neurocognitive and linguistic deficits were the most important reasons that only 59% of all inpatients completed the screening procedure. The workflow was implemented with high fidelity (96% across all steps) and proved clinically appropriate for the population, with one in five screened patients with epilepsy receiving a psychiatric diagnosis and a personalized treatment plan based on the brief, tailored psychotherapeutic intervention (*n* = 41). Fifteen of these patients (37%) had not been diagnosed previously. After 12 months, 17 patients (41%) were lost to follow‐up; this group showed significantly higher baseline depression scores. Of the 24 patients with complete follow‐up data, 17 (71%) had initiated the recommended treatment. Eleven of those who had started treatment (65%) showed reliable improvements in at least one outcome, whereas no improvements were observed in non‐adherent patients.

**Significance:**

The integrated workflow was implemented with high fidelity and was associated with promising outcomes. However, the findings highlight the need for structural reforms to improve access and effectiveness for patients with cognitive impairment, language barriers, and severe depressive symptoms.

**Key points**

- This study evaluated the implementation and clinical effectiveness of a mental health care workflow for inpatients with epilepsy.
- Due to neurocognitive and language barriers only 59% of all inpatients completed the screening procedure.
- One in five screened epilepsy patients received a psychiatric diagnosis and a personalized treatment plan.
- Sixty‐five percent of treatment‐adherent patients showed a reliable improvement in at least one outcome.
- Findings support the need to overcome systemic barriers to mental health care access in epilepsy.

## 1 INTRODUCTION

Psychiatric comorbidity is common in epilepsy and has been associated with a poor response to medical treatment, adverse psychosocial outcomes, as well as increased morbidity and mortality.^1^ Its early identification and treatment are strongly recommended by the International League Against Epilepsy (ILAE).^2^, ^3^ Epidemiological data indicate that 23% of people with epilepsy experience depression, and 20% have an anxiety disorder.^1^ However, the literature suggests that anxiety disorders and depression remain underdiagnosed and undertreated in routine clinical practice at high rates.^4^ A survey by the ILAE highlighted the lack of standardized procedures in epilepsy care settings as one of the major barriers to mental health care.^5^ Reflecting this, the inclusion of routine screening for anxiety and depression has been included in epilepsy quality measurement sets and guidelines.^6^, ^7^ Indeed, the use of an epilepsy‐specific screening measure for depression, the Neurological Disorders Depression Inventory for Epilepsy (NDDI‐E^8^) has significantly improved the detection rate of depression in busy clinical practice settings.^9^ For anxiety, the brief Epilepsy Anxiety Survey Instrument (brEASI) has been developed as an epilepsy‐specific screening measure.^10^ Although implementing systematic screening requires relatively few resources, the implementation of systematic diagnostic procedures and treatment that should follow the initial screening demands greater human resources. Furthermore, there have been few consistent outcome studies of integrated mental health services at epilepsy centers to date.^11^ A preliminary implementation study from our center demonstrated the feasibility of screening and psychotherapeutic assessment procedures, although it was limited by a relatively short study period, small sample size, lack of outcome measures, and a clinically more heterogeneous study population.^12^ The present study aims to address these limitations by evaluating our comprehensive mental health care approach with a larger and more homogeneous sample (restricted to patients with epilepsy and comorbid anxiety and/or depressive disorders, in contrast to the previous study, which included patients with dissociative seizures and other causes of transient loss of consciousness), an extended follow‐up period, and defined outcome measures. This dual‐focus design allows for the simultaneous evaluation of real‐world implementation and clinical outcomes.

## 2 METHODS

### 2.1 Setting and recruitment

This is a prospective evaluation of a clinical service implemented at a Level 4 epilepsy center (Ruhr‐Epileptology) in Germany. Recruitment took place between March 2022 and April 2023. Implementation was assessed across all consecutive inpatient admissions with a final diagnosis of epilepsy. For the effectiveness analysis, we recruited German‐speaking epilepsy inpatients with a current comorbid diagnosis of an anxiety and/or depressive disorder. Admissions to the center include both planned (elective, e.g., for presurgical evaluation) and unplanned cases via the emergency department.

### 2.2 Ethical aspects

This study was approved by the ethics committee of the Ruhr University Bochum (20‐7127). All patients provided written informed consent before participation.

### 2.3 Screening procedures

Routine screening for psychiatric comorbidities included the validated German version^13^, ^14^, ^15^ of the paper‐based NDDI‐E (cutoff value of >13^16^) and brEASI (cutoff value of >5^15^), which were presented in printed form to all eligible adult inpatients upon admission. As it is often not yet clear at the time of admission whether a final diagnosis of epilepsy will be established, all patients admitted to the epilepsy center were screened initially. For patients without a prior diagnosis of epilepsy, the 7‐item Generalized Anxiety Disorder (GAD‐7) scale without epilepsy‐specific items was used instead of the brEASI (cutoff value of >5^15^). Ineligibility criteria included all conditions that prevented individuals from reading and completing the questionnaires independently, including acute syndromes of neurocognitive dysfunction (e.g., alcohol withdrawal, delirium, status epilepticus), cognitive impairment (e.g., in patients with intellectual disability, infantile or traumatic brain damage, or dementia), and insufficient German‐language skills.

### 2.4 Diagnostic interview

All patients whose scores exceeded a cutoff value (NDDI‐E >13 and/or brEASI >5 and/or GAD‐7 >5) underwent a standardized psychiatric interview^17^ aimed at diagnostic evaluation of lifetime and current symptoms of anxiety disorders, affective disorders, adjustment disorder, posttraumatic stress disorder, and suicidality.^17^, ^18^ The diagnostic interviews were conducted by licensed psychotherapists (R.M., S.S.), who also delivered the subsequent in‐house psychotherapeutic intervention.

### 2.5 In‐house multi‐component psychotherapeutic intervention

A structured yet flexible approach ensured that evidence‐based care, individual clinical factors, and patient priorities were integrated into a coherent, actionable plan. This intervention was delivered by the same licensed psychotherapist (R.M., S.S.) who had conducted the psychiatric interview. Each session lasted ~50 min, with one or two sessions per patient. The intervention was delivered during the inpatient stay, usually within 5 days after completion of the screening. In all cases, three core components were included (see Table 1 for detailed description):

- Psychoeducation
- Information about guideline‐based treatment options
- Shared decision‐making

**TABLE 1:** Components of in‐house psychotherapeutic intervention.

*In‐house multi‐component psychotherapeutic intervention – core components*
**Psychoeducation:** Especially in psychiatric disorders, the disorder‐inherent cognitive distortion in symptom perception (such as feeling ashamed about reduced drive) may contribute to a vicious cycle that exacerbates the condition. Framing these symptoms as treatable manifestations of an illness can therefore be relieving and instill a sense of hope. Therefore, whenever the structured diagnostic interview indicated a depressive episode and/or an anxiety disorder, the therapists provided the patient with a lay‐appropriate explanation of the diagnosis.
**Information about guideline‐based treatment options:** The German guidelines for the first‐line treatment of depressive and anxiety disorders take patient preferences into account. For example, either pharmacotherapy or psychotherapeutic treatment is recommended for moderate depressive episodes and anxiety disorders depending on patient preference.^7^ Drawing on these guidelines, the therapist outlined evidence‐based treatment options appropriate for the patient's symptom severity and clinical profile, including information on the average waiting time for outpatient psychotherapy in Germany.
**Shared decision‐making:** Treatment planning was framed explicitly as a shared decision‐making process that balanced guideline recommendations with each patient's individual circumstances and personal preferences. Elements of motivational interviewing were integrated to support this process: patients were encouraged to articulate potential barriers to treatment as well as their own motivating factors, with the aim of strengthening commitment and enhancing intrinsic motivation for the agreed‐upon treatment.
*In‐house multi‐component psychotherapeutic intervention – optional components*
**Self‐help guidance:** Patients received information on evidence‐based self‐management strategies, including, for example, seizure‐specific workbooks suitable for stand‐alone use or as adjuncts in psychotherapeutic treatment.^19^, ^20^
**Support implementation:** Patients received tailored resources to implement the next steps for the modality (or combination) selected (e.g., identifying suitable general mental health care providers, a psychotherapist, psychiatrist, psychiatric clinic). With the patient's consent, these providers could contact the epilepsy center's staff for case‐related epilepsy‐specific questions. The self‐help workbooks provided as part of the “self‐help guidance” component were also designed as epilepsy‐specific adjuncts to outpatient psychotherapy.
**Concrete Implementation**: If a patient wanted to start antidepressant pharmacotherapy, they were offered commencement of treatment during their current hospital stay. Furthermore, psychiatric comorbidities were considered in interdisciplinary case conferences when optimal antiseizure medication (ASM) treatment options were discussed, for example, by switching to ASMs less closely associated with psychiatric side effects.^21^, ^22^, ^23^

Three optional components were applied as patient‐tailored elements depending on the decisions made during the shared decision‐making process and the resulting individualized treatment plan:

- Self‐help guidance
- Support implementation (follow‐up with mental health care providers)
- Concrete implementation (pharmacotherapy)

### 2.6 Follow‐up

The follow‐up assessments were conducted by the same psychotherapists who had delivered the in‐house intervention. Patients were first contacted by email at 1, 3, 6, and 12 months post‐discharge to arrange a telephone appointment; if they did not respond, at least one attempt was made to reach them directly by phone. If these contact attempts were unsuccessful, a letter was sent by mail inviting them to schedule a follow‐up conversation. Phone calls followed a semi‐structured format inquiring about their mental health, treatment plan adherence, and seizure frequency. Challenges with adherence to the treatment plan were addressed by refreshing the advice provided during the in‐house psychotherapeutic intervention.

### 2.7 Implementation and clinical effectiveness outcome measures

The study quantified key implementation constructs according to the taxonomy proposed by Proctor et al. to capture how successfully the workflow was delivered in routine inpatient care.^24^ “Penetration” refers to the degree of integration of an intervention within a service system and was assessed at the level of workflow entry (“initial step penetration”), operationalized as the proportion of screened patients among all inpatient admissions with an epilepsy diagnosis. In the present study, this assessment did not include patients with predominantly dissociative seizures and comorbid epilepsy, as these patients were referred to a treatment pathway designed specifically for dissociative seizures (irrespective of screening scores). “Fidelity” of implementation denotes the extent to which it was delivered as intended, and was first quantified for each step of the workflow separately: proportion of eligible patients screened; proportion of positively screened patients undergoing diagnostic interview; and proportion of diagnosed patients completing the in‐house psychotherapeutic intervention. In addition, a composite fidelity score for the entire workflow was calculated (total completed steps/total applicable steps). Finally, the “appropriateness” of the workflow reflects the perceived fit for the target population, and was judged by the rate of new psychiatric diagnoses and the completion of indicated in‐house psychotherapeutic interventions in the workflow cohort.

As part of the effectiveness study, patients completed questionnaires online or paper‐based at baseline, after 1, 3, 6, and 12 months. Baseline was defined as the timepoint immediately following the diagnostic interview, that is, after the confirmed diagnosis of an anxiety and/or depressive disorder and the patient's informed consent to participate. The primary outcome measure was health‐related quality of life at 12 months, assessed using the Quality of Life Inventory in Epilepsy (QOLIE‐31) total score. Secondary outcomes included the Beck Depression Inventory II (BDI‐II), the Beck Anxiety Inventory (BAI), and the Work and Social Adjustment Scale (WSAS). The QOLIE‐31 is a widely established epilepsy‐specific questionnaire for recording health‐related quality of life (HRQoL) with 31 items.^25^ The BDI‐II is a self‐report questionnaire for the assessment of depressive symptom severity with 21 items^26^, ^27^; the BAI assesses the presence and severity of anxiety symptoms with 21 items.^28^ The WSAS is used widely across mental health and neurological populations, designed to assess functional impairment in five domains: work, home management, social leisure activities, private leisure activities, and close relationships.^29^ Seizure frequency was assessed as a secondary outcome at baseline, after 1, 3, 6, and 12 months, and categorized as: ≥1 seizure/day, ≥1/week, ≥1/month, <1/month.

### 2.8 Data analysis

Both Microsoft Excel and IBM SPSS Statistics 21 software were used to analyze the data. Individual‐level change was quantified using the Reliable Change Index (RCI),^30^ which is recommended for uncontrolled designs with small samples.^31^ It is calculated based on individual patient data, allowing determination whether observed score changes exceed measurement error, that is, the proportion of patients who have reliably improved (RCI+), worsened (RCI–) or not changed (RCI0). If the RCI is greater than 1.96 or less than −1.96, this is a statistically reliable change at the 95% confidence level. We also indicate how many patients with unchanged RCIs of BDI‐II and BAI questionnaires reported mild depressive or anxiety symptoms (BDI‐II ≤13, BAI ≤7) at baseline to facilitate the interpretation of these unchanged RCIs. Analyses were conducted on completers only; thus, results do not represent an intention‐to‐treat analysis. No imputation of missing data was performed.

To analyze mean differences, one‐tailed paired *t* tests were conducted. A significance level of *p* < .05 was applied. The Bonferroni–Holm correction was used to correct for multiple comparisons. The relationship between treatment adherence and RCIs for psychiatric symptom severity (BDI‐II, BAI) HRQoL (QOLIE‐31 and WSAS total score) was analyzed descriptively.

Any change of the pre‐defined seizure frequency category was considered a relevant change. Penetration, adoption, and fidelity were quantified as simple proportions (see above).

To examine potential selection biases, patients who completed the 12‐month follow‐up and those who did not were compared regarding age, gender, BDI‐II, BAI, QOLIE‐31, and WSAS scores. Continuous variables were analyzed using independent‐sample *t* tests, and categorical variables using chi‐square tests. Significance was set at *p* < .05.

## 3 RESULTS

### 3.1 Implementation outcomes

**FIGURE 1:**
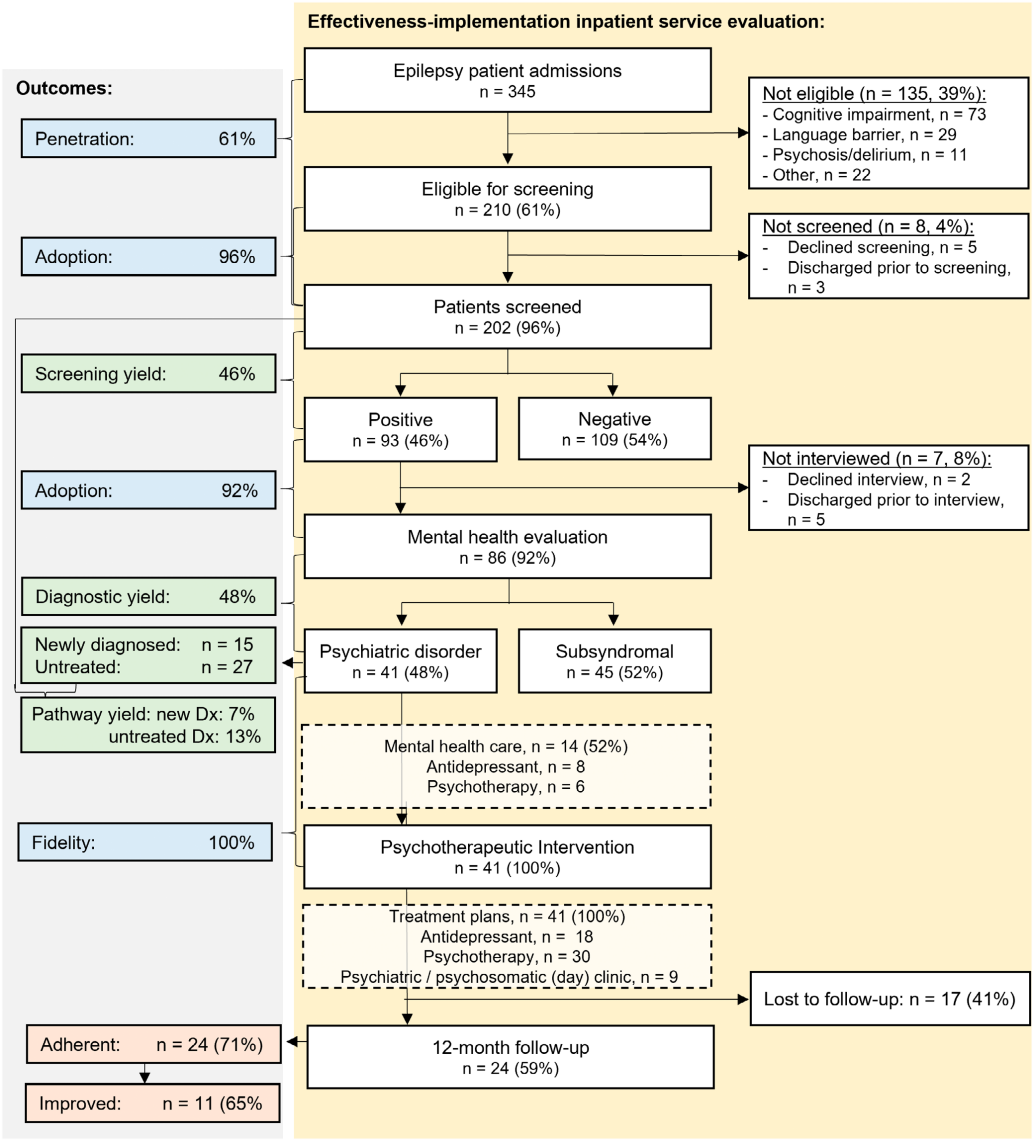
Mental health care workflow. Illustration of the entire mental health care workflow. Dx, diagnoses.

A total of 454 inpatients were admitted to the epilepsy center during the recruitment period. About half of these patients were planned (elective cases) and half of them unplanned, that is, admitted via the emergency department. One hundred five patients (23%) ultimately did not receive an epilepsy diagnosis and four patients had predominantly dissociative seizures with comorbid epilepsy, leaving 345 epilepsy inpatients who were candidates for inclusion in the implementation analysis. Of these, 210 of epilepsy patients (61%) were eligible for screening. Although fidelity of screening was excellent at 96% (202 screened of 210 eligible), initial step penetration was 59% (202/345) with the major factor being ineligibility. A diagnostic interview was conducted with 86 of all 93 of 202 patients with screening scores above clinical cutoffs (screening yield: 46%), reflecting a fidelity score for the diagnostic interview of 92%. Of these 86 patients with epilepsy who were interviewed, 41 (48%) received a diagnosis of depression and/or anxiety disorder. New diagnoses thus constituted 7% (15/210) of the total number of patients entering the workflow, and 17% (15/86) of those interviewed, strongly supporting the “appropriateness” of the workflow. The fidelity of the in‐house psychotherapeutic intervention was 100%, as it was completed per protocol in all 41 diagnosed patients. As an additional measure of appropriateness, 20% (41/210) of patients entering the workflow received an indicated psychotherapeutic intervention leading to the development of a personalized treatment plan. The composite fidelity score of the entire workflow revealed that 96% of all applicable steps had been completed per protocol [(202 + 86 + 41)/(210 + 93 + 41)]. The entire mental health care workflow is illustrated in Figure 1.

### 3.2 Characteristics of patients with epilepsy and psychiatric comorbidity

Table 2 summarizes the characteristics of the final sample of 41 patients with epilepsy and psychiatric comorbidity. The majority of patients lived with longstanding pharmacoresistant epilepsy. The rate of undiagnosed comorbidity was higher in anxiety disorders (10/16, 63%) than in depression (6/31, 19%).

**TABLE 2:** Characteristics of patients with epilepsy and psychiatric comorbidity (*n* = 41).

Characteristic	*n* (%)	Median (range)
Sex		
Female	25 (61%)	
Male	16 (39%)	
Age, years		49 (21–81)
Epilepsy type		
Focal epilepsy	28 (68%)	
Generalized epilepsy	10 (24%)	
Combined focal and generalized	1 (2%)	
Unknown type	2 (5%)	
Pharmacoresistant epilepsy	30 (73%)	
Duration of epilepsy, years		16 (0–62)
Newly diagnosed epilepsy	7 (17%)	
Psychiatric comorbidities		
Depression	31 (76%)	
—of which previously undiagnosed	6 (19%)	
Depressive symptom severity (BDI‐II)		
Minimal (≤13)	7 (17.1%)	
Mild (14–19)	10 (24.4%)	
Moderate (20–28)	12 (29.3%)	
Severe (≥29)	11 (26.8%)	
Anxiety disorder	16 (39%)	
—of which previously undiagnosed	10 (63%)	
Anxiety symptom severity (BAI)		
Minimal (≤7)	4 (9.8%)	
Mild (8–15)	14 (34.1%)	
Moderate (16–25)	11 (26.8%)	
Severe (≥26)	11 (26.8%)	

Abbreviations: BAI, Beck Anxiety Inventory; BDI‐II, Beck Depression Inventory II.

### 3.3 Treatment plans

Overall, 27 of all 41 participating patients (66%) did not receive any mental health care at baseline. Even among the 26 patients with a prior psychiatric diagnosis, only 14 patients (52%) were already receiving guideline‐based care (see Figure 1 for baseline treatment and treatment plans following the in‐house intervention).

### 3.4 Treatment adherence after 12 months

After 12 months, follow‐up data were available for 24 of 41 patients (59%). Of these, 17 patients (71%) had successfully initiated at least one recommended treatment: Ten patients were currently undergoing or had completed outpatient psychotherapy, eight patients were receiving antidepressant pharmacotherapy, and one patient had received inpatient treatment in a psychiatric clinic. Two patients (8%) were still on a waiting list for outpatient psychotherapy and five patients (21%) had not initiated any form of treatment. In addition, ASMs had been changed in 18 patients (75%) and one patient had undergone epilepsy surgery. An exploratory comparison between patients who completed follow‐up after 12 months (*n* = 24) and those lost to follow‐up (*n* = 17) revealed significantly higher mean baseline BDI‐II scores (28.25 vs 19.92; *p* = .025), indicating more severe depressive symptoms at baseline.

### 3.5 Treatment adherence and reliable outcome changes

**FIGURE 2:**
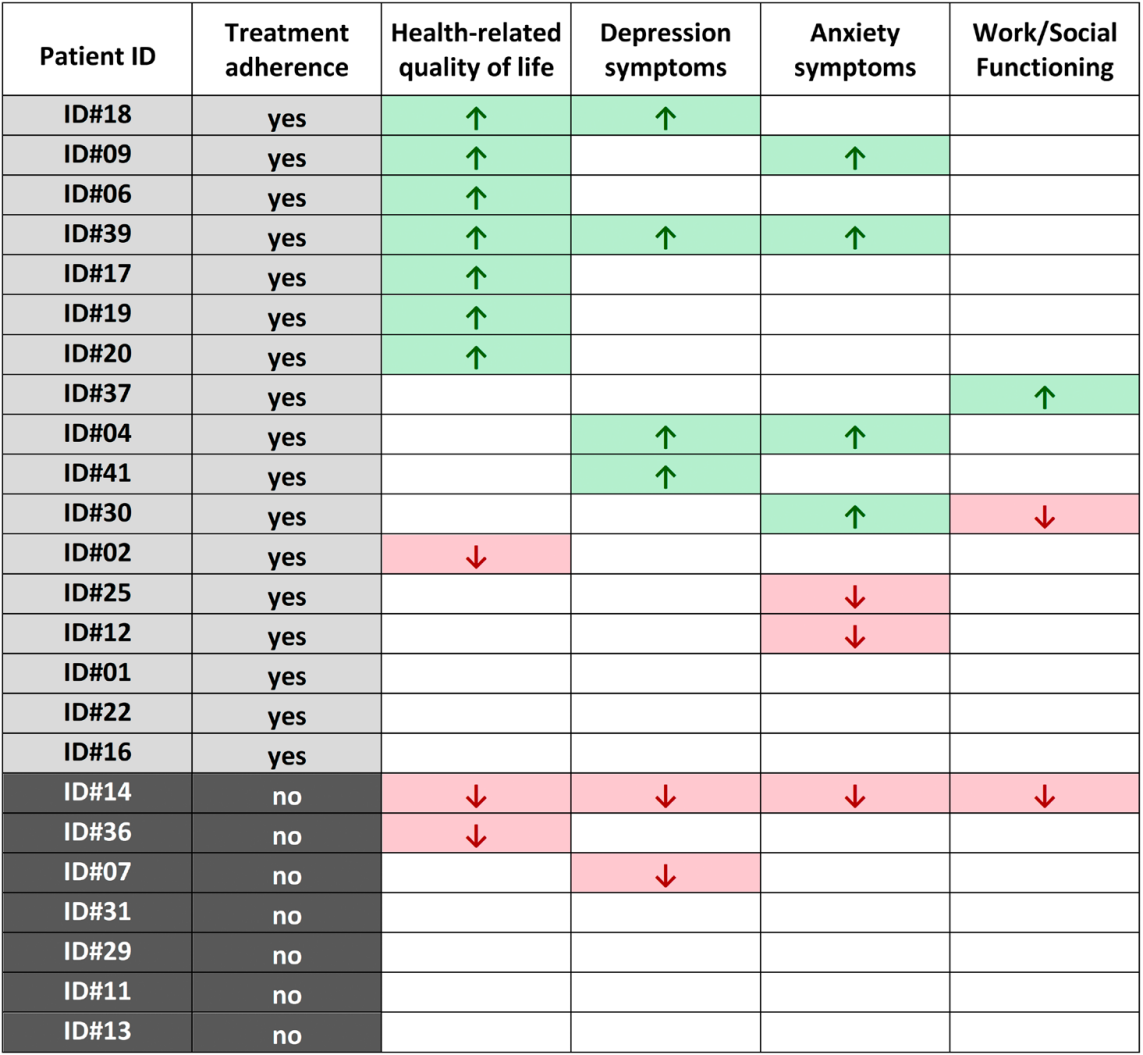
Treatment adherence and reliable outcome changes (RCI) at 12‐month follow‐up. Each row represents one participant. Arrows indicate reliable improvement (RCI+, ↑) or deterioration (RCI–, ↓) according to the RCI in each outcome domain. White cells represent no reliable change (RCI0). “Yes” denotes treatment adherence, “No” denotes non‐adherence.

After 12 months, 17 of 24 patients (71%) had successfully initiated at least one recommended treatment. A total of 11 of 24 patients (46%) showed reliable improvement (RCI+) in at least one outcome. All of these 11 patients were adherent to therapy. In summary, none of the 7 patients who had not yet implemented any form of treatment showed any improvements (RCI0, RCI–), whereas 11 of 17 adherent patients (65%) showed a reliable improvement (RCI+) in at least one outcome (see Figure 2 and Tables [Link], [Link]).

### 3.6 Health‐related quality of life

After 12 months, 14 of 24 patients (58%) showed no improvement (RC0) in the QOLIE‐31 total score, 7 of 24 patients (29%) demonstrated reliable improvement (RCI+), and 3 of 24 patients (13%) showed a deterioration (RCI–); 21 of 24 patients (88%) showed no improvement (RCI0) of the WSAS score, 1 of 24 patients (4%) demonstrated reliable improvement (RCI+), and 2 of 24 patients (8%) showed a deterioration (RCI–) (see Tables S6 and S7 for all other survey dates). No significant changes in mean QOLIE‐31 or WSAS scores were observed across the group at 1, 3, 6, or 12 months (Table 3).

**TABLE 3:** Mean differences were analyzed after 1 month (*M*_1_, *N* = 31), and 3 (*M*_3_, *N* = 28), 6 (*M*_6_, *N* = 27), and 12 months (*M*_12_, *N* = 24) using one‐tailed paired *t* tests.

	*M*_B_	*M*_1_	*t*	*p*(BH)	*M*_3_	*t*	*p*(BH)	*M*_6_	*t*	*p*(BH)	*M*_12_		*p*(BH)
WSAS Total Score	16.5	15.5	.506	.930	15.6	1.168	.520	15.3	−.307	.930	15.4	−.369	.930
QOLIE‐31 Total Score	44.9	46.5	−.819	>.999	49.8	−2.956	.096	50.0	−1.404	>.999	51.6	−1.657	>.999
Seizure Worry	37.9	38.1	−.038	>.999	40.3	−1.493	>.999	42.2	−.913	>.999	47.0	−1.778	>.999
Overall QoL	49.4	47.9	.43	>.999	51.1	.260	>.999	53.3	−.718	>.999	50.9	.037	>.999
Emotional Well‐being	45.4	50.3	−1.548	>.999	49.7	−1.731	>.999	53.5	−2.328	.420	52.5	−.780	>.999
Energy/Fatigue	35.7	39.5	−1.187	>.999	41.4	−1.782	>.999	41.5	−1.081	>.999	43.5	−2.333	.421
Cognition	42.9	44.2	−.45	>.999	50.6	−1.760	>.999	48.6	−.127	>.999	49.5	.893	>.999
Medication Effects	44.9	46.1	−.212	>.999	47.5	−.737	>.999	50.9	−.492	>.999	54.6	−1.571	>.999
Social Function	51.9	53.0	−.305	>.999	56.9	−2.598	.233	55.0	−.555	>.999	59.9	−1.735	>.999

*Note*: The Bonferroni–Holm correction was used to correct for multiple comparisons.

Abbreviations: *M*_1_/*M*_3_/*M*_6_/*M*_12_, mean at 1/3/6/12 month(s); *M*_B_, mean at baseline; QoL, Quality of Life; QOLIE‐31, Quality of Life Inventory in Epilepsy with 31 items; WSAS, Work and Social Adjustment Scale.

With regard to QOLIE‐31 subscales, the highest proportion of reliable improvement (RCI+) among patients was found in the subscales “Emotional Well‐being” (7/24; 29%) and “General Quality of Life” (6/24; 25%). The lowest improvement rates (RCI+) were found in “Cognition” and “Social Functioning” (2/24; 8% each). The highest rate of deterioration (RCI–) occurred in the subscale “General Quality of Life” (6/24; 25%) (Table S6). No statistically significant improvements were shown on *t* tests in any QOLIE‐31 subscale scores between baseline and follow‐up assessments across the group (Table 3).

### 3.7 Depressive and anxiety symptom severity

Twelve months after baseline measurement, 13 of 24 patients (54%) showed no improvement (RCI0) in BDI‐II scores. In 5 of 24 patients (21%), depressive symptom severity remained very mild (RCI0) (BDI‐II ≤13) and 4 of 24 patients (17%) showed a reliable improvement (RCI+), whereas scores in 2 of 24 patients (8%) showed a deterioration (see Table S8 for all other survey dates). Paired *t* tests showed no significant decrease in BDI‐II scores for the entire group at 1 month or at 12 months. However, significant reductions were observed at 3 months (*t*(27) = 2.823, *p* = .018) and 6 months (*t*(26) = 2.711, p = .018), indicating short‐ to mid‐term improvements (Table 4). Twelve months post‐baseline, 15 of 24 patients (63%) patients showed no improvement (RCI0) of BAI scores and 4 of 24 patients (17%) showed a reliable improvement (RCI+); in 2/24 patients (8%), anxiety symptoms remained very mild (RCI0) (BAI ≤7) and 3/24 patients (12%) showed a deterioration (RCI–) (see Table S8 for all other survey dates). No significant changes in BAI scores were observed across the group at 1, 3, 6, or 12 months (Table 4).

**TABLE 4:** Mean differences were analyzed after 1 month (*M*_1_, *N* = 31), and 3 (*M*_3_, *N* = 28), 6 (*M*_6_, *N* = 27), and 12 months (*M*_12_, *N* = 24) using one‐tailed paired *t* tests.

	*M*_B_	*M*_1_	*t*	*p*(BH)	*M*_3_	*t*	*p*(BH)	*M*_6_	*t*	*p*(BH)	*M*_12_	*t*	*p*(BH)
BDI	23.8	23.1	.433	.334	18.4	2.823	.018*	17.0	2.711	.018*	16.7	1.575	.130
BAI	22.2	20.0	1.121	.271	17.6	1.504	.216	14.7	1.930	.130	15.6	1.090	.271

*Note*: The Bonferroni–Holm correction was used to correct for multiple comparisons.

Abbreviations: BAI, Beck Anxiety Inventory; BDI, Beck Depression Inventory II; *M*_1_/*M*_3_/*M*_6_/*M*_12_, mean at 1/3/6/12 month(s); *M*_B_, mean at baseline.

* *p* < .05.

### 3.8 Seizure frequency

After 12 months, 11 of 24 patients (46%) reported a relevant improvement of their seizure frequency; at least one improved RCI was found in six of these patients (55%) (Table S9). No relevant changes of seizure frequency were reported by 9 of 24 patients (37%). In 3 of 24 patients (13%), seizure frequency remained in the lowest pre‐defined category “<1/month.” A relevant increase in seizures was observed by one patient.

## 4 DISCUSSION

The implementation analysis revealed that our integrated mental health workflow for inpatients with epilepsy was appropriate and feasibly employed with high fidelity, but limited penetration: 39% of patients were not eligible to enter the algorithmic workflow, largely due to cognitive and language barriers as well as acute morbidity. This highlights a critical gap in mental health care access, made worse by the fact that the very characteristics preventing inclusion—neurocognitive deficits and linguistic marginalization—are at the same time strong predisposing factors for psychiatric morbidity.^32^, ^33^ This issue is particularly salient in specialized epilepsy centers, where individuals with cognitive impairment are overrepresented due to the shared etiologies of pharmacoresistant epilepsy and developmental brain disorders or traumatic brain injury.^34^ As a result, this constitutes a large and underserved subgroup within the epilepsy population.^35^ Addressing these barriers would require the use of adapted, simplified screening tools—for instance, the German version of the Glasgow Depression Scale for people with a Learning Disability (GDS‐LD)^36^—and the allocation of additional personnel resources to involve caregivers, deliver adapted psychosocial interventions, and conduct assessments and interventions assisted by translators.^35^ In line with existing literature, the study revealed a high rate of previously undetected psychiatric comorbidities: 37% of participants had a previously undiagnosed depressive and/or anxiety disorder. This rate aligns with prior research indicating that depression and anxiety disorders often remain unrecognized in epilepsy care.^4^ A closer analysis of diagnostic status within our sample, however, revealed a more nuanced picture: although 81% of depressive disorders had already been diagnosed prior to study participation, 63% of anxiety disorders had remained unrecognized. Compared to the rates of underdiagnosis reported in the literature—33% for depression and 64% for anxiety disorders^4^—our findings highlight that the rate of undiagnosed anxiety disorders remains alarmingly high. This discrepancy underscores the particular diagnostic challenge anxiety symptoms pose in epilepsy care and the continued need for increased clinical awareness and standard screening.^6^, ^7^

Although the majority of patients who adhered to the recommended treatments showed reliable improvements, the absence of such improvements in a substantial subgroup underscores important limitations of existing mental health care approaches for people with epilepsy. Our response rate is comparable to response rates in patients without epilepsy^37^; however, they nevertheless suggest that current guideline‐based interventions might be insufficient to address the complex neuropsychiatric profiles frequently seen in epilepsy.

Conversely, none of the patients who had not implemented any part of the recommended treatment plan showed any improved outcomes. This reinforces the central importance of treatment uptake and suggests that systemic barriers to access may be a decisive limiting factor, particularly for individuals with reduced drive and mobility due to their condition.

Although 46% of patients reported a relevant improvement in seizure frequency after 12 months, only half of them showed a corresponding improvement in RCI, in line with the view that seizure reduction alone does not necessarily translate into improvements in psychiatric symptoms or HRQoL.^1^ This underscores the need for a comprehensive therapeutic approach in patients with epilepsy and psychiatric comorbidities.

Of note, patients lost to follow‐up had significantly higher baseline depressive symptom severity than patients who completed follow‐up. This finding suggests that depressive symptoms may have interfered with the implementation of recommended treatments. These patients might have benefited from a more immediate and structured referral to mental health services during the inpatient stay.

Reviews by the ILAE Psychology Task Force and ILAE Psychiatry Commission have identified multiple international mental health care models of integrated care that show promise in improving uptake, continuity, and outcomes of mental health care in epilepsy.^11^, ^38^ Successful mental health care models often include routine screening, on‐site mental health professionals, and clearly defined referral pathways. These elements were partially implemented in the present evaluated service. However, the effectiveness of this particular service would benefit from further structural support—such as on‐site referral pathways to psychiatrists and psychotherapists, including liaison services in psychosomatic medicine and psychiatry—and policy‐level support, including reimbursement frameworks that facilitate outpatient psychotherapeutic and psychiatric treatment initiation by neurological clinics.

## 5 LIMITATIONS

This study has several limitations that must be considered. First, we did not include screening instruments adapted for patients with cognitive impairments or intellectual disabilities. This was due primarily to the study's focus on evaluating the implementation of *self‐report* screening tools (i.e., no additional personnel resources had been allocated to support patients in questionnaire completion or involvement of carers). Consequently, a substantial proportion of patients had to be excluded from screening. This exclusion was unexpected and, in our view, represents one of the most important findings of the study. It underscores the urgent need for future research to include adapted screening instruments and resource planning for this underserved patient group. Second, the high dropout rate (41%) reduces the statistical power of the follow‐up analyses. Because analyses were restricted to participants who completed the 12‐month follow‐up, findings may be subject to attrition bias and should not be interpreted as intention‐to‐treat results. Future studies with larger cohorts should apply longitudinal modeling and multiple‐imputation techniques to validate and extend these findings. Third, this study is uncontrolled, which limits causal inference. However, randomization on the individual level would be ethically problematic in this context, as it would require withholding already established guideline‐conform care from our patients.^12^ Therefore, larger prospective cohort studies and cluster‐randomized trials^39^ may represent ethically sound and methodologically robust alternatives for future research. Future research should apply the methodological rigor of pre‐registration and reporting compliant with Consolidated Standards of Reporting Trials (CONSORT), to allow for stronger causal inference and external validity and to incorporate participatory trial design principles to strengthen patient involvement. In addition, seizure frequencies were self‐reported and may therefore be unreliable, as prior research has shown that patient‐reported seizure frequency often lacks reliability.^40^ Nonetheless, given the accumulating evidence that untreated psychiatric comorbidities increase the risk of pharmacoresistant epilepsy,^41^ future research should explore whether integrated mental health care contributes to improved seizure control. Moreover, adherence data were also based on patient self‐report, defined as engagement in follow‐up care with specialist clinics, psychiatrists, or licensed psychotherapists. These reports were not independently verified, which may limit the accuracy of adherence and quality assessment. Finally, it should be noted that the study population consisted predominantly of inpatients with long‐standing epilepsy. Consequently, the findings may not be directly generalizable to outpatient populations, who may experience different psychosocial circumstances and treatment trajectories.

## 6 CONCLUSIONS AND OUTLOOK

Considering the high fidelity of implementation, the substantial rate of new psychiatric diagnoses, and the reliable improvements observed in adherent patients, the integration of standardized mental health care into epilepsy services appears to represent a worthwhile use of resources in specialized epilepsy settings. However, access barriers (including cognitive impairment, language barriers, and acute morbidity) and the lack of improvement in some adherent patients demonstrate that current treatment options within the health care system are not yet sufficient to meet the needs of this complex patient group. Further development of effective treatment methods and structural reforms toward more inclusive and better‐integrated care pathways appear warranted—ideally involving carers, translators, and low‐threshold access to mental health care specialists to facilitate assessments and interventions in patients facing disorder‐related (e.g., cognitive, affective) or language‐related barriers.

## AUTHOR CONTRIBUTIONS

Study conception and design: Rosa Michaelis, Markus Reuber, and Stoyan Popkirov. Data acquisition: Rosa Michaelis, Dorothea Hölscher, Katharina Braun, Sabine Schlömer, Wenke Grönheit, Tim Wehner, and Claudia Grunert. Data analysis and interpretation: Rosa Michaelis, Dorothea Hölscher, Katharina Braun, Johannes Jungilligens, and Stoyan Popkirov. Writing – original draft: Rosa Michaelis and Stoyan Popkirov. Writing – review and editing: Dorothea Hölscher, Katharina Braun, Sabine Schlömer, Wenke Grönheit, Tim Wehner, Claudia Grunert, Markus Reuber, Johannes Jungilligens, Jörg Wellmer, Friedrich Edelhäuser, and Corinna Seliger.

## FUNDING INFORMATION

R.M.'s scientific work was funded by the internal FoRUM funding program (project K160‐20‐A) of the Faculty of Medicine at the Ruhr University Bochum. S.P. is supported by an Advanced Clinician Scientist Programme UMEA^2^ (01EO2104) from the German Federal Ministry of Education and Research (BMBF).

## CONFLICT OF INTEREST STATEMENT

Rosa Michaelis received royalties for the German seizure‐specific treatment workbooks published by Pabst and Hippocampus. None of the authors has any conflict of interest to disclose.

## ETHICS STATEMENT

This study was approved by the ethics committee of the Ruhr University Bochum (20‐7127). We confirm that we have read the Journal's position on issues involved in ethical publication and affirm that this report is consistent with those guidelines.

## PATIENT CONSENT STATEMENT

All patients provided written informed consent before participation.

## Supporting information


Table S1.

Table S2.

Table S6.

Table S8.

Table S9.

## Data Availability

Data are available upon reasonable request from the corresponding author.
