# Supplementary material for: Effectiveness and implementation of an inpatient mental health care pathway at an epilepsy center: A prospective service evaluation
Source: Epilepsia. 2025 Nov 14;67(3):1358–70. doi: 10.1111/epi.70014 (PMC13007824; doi:10.1111/epi.70014)
Supplement: Supplementary file 1 — Table S1. [file EPI-67-1358-s004.docx]

**Supplementary Table 1**

| **Patient ID** | **Treatment adherence** | **QOLIE31 baseline** | **QOLIE31_12m** | **BDI_baseline** | **BDI_12m** | **BAI_baseline** | **BAI_12m** | **ASAS baseline** | **ASAS_12m** |
| --- | --- | --- | --- | --- | --- | --- | --- | --- | --- |
| **ID#18** | **yes** | 41,06 | 69 | 28 | 5 | 15 | 11 | 8 | 0 |
| **ID#09** | **yes** | 57,22 | 66,4 | 11 | 8 | 17 | 6 | 9 | 6 |
| **ID#06** | **yes** | 46,32 | 75,87 | 14 | 10 | 17 | 17 | 2 | 0 |
| **ID#39** | **yes** | 48,67 | 61,64 | 35 | 15 | 42 | 31 | 13 | 8 |
| **ID#17** | **yes** | 33,32 | 46,37 | 25 | 24 | 14 | 12 | 14 | 9 |
| **ID#19** | **yes** | 59,92 | 74,29 | 13 | 19 | 14 | 16 | 6 | 8 |
| **ID#20** | **yes** | 61,94 | 76,35 | 4 | 0 | 10 | 3 | 2 | 2 |
| **ID#37** | **yes** | 48,81 | 50,27 | 16 | 13 | 22 | 18 | 26 | 15 |
| **ID#04** | **yes** | 29,54 | 27,36 | 38 | 11 | 38 | 6 | 24 | 35 |
| **ID#41** | **yes** | 55,06 | 58,37 | 29 | 13 | 8 | 2 | 13 | 15 |
| **ID#30** | **yes** | 71,56 | 70,9 | 2 | 6 | 14 | 3 | 6 | 12 |
| **ID#02** | **yes** | 40,42 | 23,52 | 22 | 26 | 25 | 29 | 31 | 30 |
| **ID#25** | **yes** | 58,24 | 51,7 | 22 | 25 | 11 | 21 | 0 | 8 |
| **ID#12** | **yes** | 46,19 | 50,43 | 18 | 20 | 18 | 36 | 11 | 10 |
| **ID#01** | **yes** | 47,01 | 51,45 | 11 | 10 | 5 | 7 | 20 | 16 |
| **ID#22** | **yes** | 44,63 | 42,65 | 19 | 15 | 9 | 12 | 14 | 15 |
| **ID#16** | **yes** | 37,79 | 45,91 | 37 | 40 | 19 | 16 | 8 | 4 |
| **ID#14** | **no** | 42,33 | 29,21 | 9 | 22 | 7 | 17 | 32 | 36 |
| **ID#36** | **no** | 48,62 | 39,84 | 21 | 14 | 21 | 19 | 5 | 20 |
| **ID#07** | **no** | 31,23 | 33,41 | 23 | 37 | 25 | 25 | 26 | 26 |
| **ID#31** | **no** | 47,6 | 47,26 | 29 | 26 | 4 | 2 | 16 | 10 |
| **ID#29** | **no** | 46,28 | 44,1 | 21 | 16 | 33 | 31 | 24 | 25 |
| **ID#11** | **no** | 51,56 | 54,38 | 17 | 16 | 25 | 20 | 27 | 34 |
| **ID#13** | **no** | 51,37 | 46,96 | 14 | 10 | 12 | 15 | 18 | 22 |
