# Supplementary material for: Effectiveness and implementation of an inpatient mental health care pathway at an epilepsy center: A prospective service evaluation
Source: Epilepsia. 2025 Nov 14;67(3):1358–70. doi: 10.1111/epi.70014 (PMC13007824; doi:10.1111/epi.70014)
Supplement: Supplementary file 2 — Table S2. [file EPI-67-1358-s005.docx]

| **Patient ID** | **Treatment adherence** | **QOLIE31**  **baseline** | **QOLIE31_1m** | **QOLIE31_3m** | **QOLIE31_6m** | **QOLIE31_12m** |
| --- | --- | --- | --- | --- | --- | --- |
| **ID#18** | **yes** | 41,06 | 56,33 | 51,85 | 66,7 | 69 |
| **ID#09** | **yes** | 57,22 | 67,79 |  | 62,23 | 66,4 |
| **ID#06** | **yes** | 46,32 | 35,27 | 55,01 | 60,69 | 75,87 |
| **ID#39** | **yes** | 48,67 | 40,99 | 72,93 | 49,93 | 61,64 |
| **ID#17** | **yes** | 33,32 | 34,97 | 49,68 | 41,62 | 46,37 |
| **ID#19** | **yes** | 59,92 | 71,97 | 60,05 | 50,95 | 74,29 |
| **ID#20** | **yes** | 61,94 |  | 61,3 | 54,25 | 76,35 |
| **ID#37** | **yes** | 48,81 | 46,25 | 56,52 | 57,23 | 50,27 |
| **ID#04** | **yes** | 29,54 | 25,08 | 23,24 | 29,72 | 27,36 |
| **ID#41** | **yes** | 55,06 | 44,64 | 35,73 | 41,22 | 58,37 |
| **ID#30** | **yes** | 71,56 | 63,29 | 67,95 | 75,11 | 70,9 |
| **ID#02** | **yes** | 40,42 | 35,9 | 41,96 | 29,97 | 23,52 |
| **ID#25** | **yes** | 58,24 | 56,23 |  | 54,58 | 51,7 |
| **ID#12** | **yes** | 46,19 | 40,93 | 43,66 | 43,89 | 50,43 |
| **ID#01** | **yes** | 47,01 | 49,81 | 61,31 | 49,21 | 51,45 |
| **ID#22** | **yes** | 44,63 | 58,69 | 53,71 | 42,71 | 42,65 |
| **ID#16** | **yes** | 37,79 | 45,68 | 47,6 | 26,17 | 45,91 |
| **ID#14** | **no** | 42,33 |  | 35,3 | 49,56 | 29,21 |
| **ID#36** | **no** | 48,62 | 38,48 | 50,14 | 41,48 | 39,84 |
| **ID#07** | **no** | 31,23 | 28,8 | 46,1 |  | 33,41 |
| **ID#31** | **no** | 47,6 | 51,78 | 58,66 | 59,71 | 47,26 |
| **ID#29** | **no** | 46,28 | 46,66 | 49,95 | 54,89 | 44,1 |
| **ID#11** | **no** | 51,56 | 65,21 | 55,29 | 43,84 | 54,38 |
| **ID#13** | **no** | 51,37 | 38,56 | 47,56 | 51,78 | 46,96 |

**Supplementary Table 2**

**Supplementary Table 3**

| **Patient ID** | **Treatment adherence** | **BDI_baseline** | **BDI_1m** | **BDI_3m** | **BDI_6m** | **BDI_12m** |
| --- | --- | --- | --- | --- | --- | --- |
| **ID#18** | **yes** | 28 | 15 | 12 | 8 | 5 |
| **ID#09** | **yes** | 11 | 11 |  | 15 | 8 |
| **ID#06** | **yes** | 14 | 21 | 8 | 12 | 10 |
| **ID#39** | **yes** | 35 | 35 | 9 | 26 | 15 |
| **ID#17** | **yes** | 25 | 23 | 24 | 26 | 24 |
| **ID#19** | **yes** | 13 | 16 | 17 | 17 | 19 |
| **ID#20** | **yes** | 4 |  | 7 | 8 | 0 |
| **ID#37** | **yes** | 16 | 22 | 6 | 6 | 13 |
| **ID#04** | **yes** | 38 | 23 | 9 | 20 | 11 |
| **ID#41** | **yes** | 29 | 24 | 33 | 27 | 13 |
| **ID#30** | **yes** | 2 | 2 | 5 | 0 | 6 |
| **ID#02** | **yes** | 22 | 20 | 20 | 21 | 26 |
| **ID#25** | **yes** | 22 | 25 |  | 26 | 25 |
| **ID#12** | **yes** | 18 | 30 | 19 | 16 | 20 |
| **ID#01** | **yes** | 11 | 10 | 12 | 7 | 10 |
| **ID#22** | **yes** | 19 | 27 | 15 | 18 | 15 |
| **ID#16** | **yes** | 37 | 40 | 31 | 30 | 40 |
| **ID#14** | **no** | 9 |  | 13 | 6 | 22 |
| **ID#36** | **no** | 21 | 18 | 14 | 11 | 14 |
| **ID#07** | **no** | 23 | 20 | 16 |  | 37 |
| **ID#31** | **no** | 29 | 27 | 28 | 10 | 26 |
| **ID#29** | **no** | 21 | 13 | 15 | 4 | 16 |
| **ID#11** | **no** | 17 | 18 | 16 | 20 | 16 |
| **ID#13** | **no** | 14 | 34 | 22 | 25 | 10 |

**Supplementary Table 4**

| **Patient ID** | **Treatment adherence** | **BAI_baseline** | **BAI_1m** | **BAI_3m** | **BAI_6m** | **BAI_12m** |
| --- | --- | --- | --- | --- | --- | --- |
| **ID#18** | **yes** | 15 | 11 | 13 | 7 | 11 |
| **ID#09** | **yes** | 17 | 11 |  | 14 | 6 |
| **ID#06** | **yes** | 17 | 25 | 5 | 10 | 17 |
| **ID#39** | **yes** | 42 | 28 | 13 | 40 | 31 |
| **ID#17** | **yes** | 14 | 19 | 15 | 14 | 12 |
| **ID#19** | **yes** | 14 | 15 | 23 | 26 | 16 |
| **ID#20** | **yes** | 10 |  | 15 | 2 | 3 |
| **ID#37** | **yes** | 22 | 32 | 17 | 16 | 18 |
| **ID#04** | **yes** | 38 | 10 | 8 | 17 | 6 |
| **ID#41** | **yes** | 8 | 18 | 13 | 9 | 2 |
| **ID#30** | **yes** | 14 | 0 | 8 | 3 | 3 |
| **ID#02** | **yes** | 25 | 26 | 29 | 21 | 29 |
| **ID#25** | **yes** | 11 | 20 |  | 20 | 21 |
| **ID#12** | **yes** | 18 | 24 | 23 | 33 | 36 |
| **ID#01** | **yes** | 5 | 5 | 8 | 5 | 7 |
| **ID#22** | **yes** | 9 | 9 | 10 | 15 | 12 |
| **ID#16** | **yes** | 19 | 17 | 28 | 17 | 16 |
| **ID#14** | **no** | 7 |  | 12 | 11 | 17 |
| **ID#36** | **no** | 21 | 20 | 10 | 13 | 19 |
| **ID#07** | **no** | 25 | 32 | 19 |  | 25 |
| **ID#31** | **no** | 4 | 6 | 3 | 2 | 2 |
| **ID#29** | **no** | 33 | 21 | 17 | 2 | 31 |
| **ID#11** | **no** | 25 | 27 | 26 | 24 | 20 |
| **ID#13** | **no** | 12 | 27 | 19 | 24 | 15 |

**Supplementary Table 5**

| **Patient ID** | **Treatment adherence** | **ASAS**  **baseline** | **ASAS_1m** | **ASAS_3m** | **ASAS_6m** | **ASAS_12m** |
| --- | --- | --- | --- | --- | --- | --- |
| **ID#18** | **yes** | 8 | 0 | 0 | 0 | 0 |
| **ID#09** | **yes** | 9 | 11 |  | 6 | 6 |
| **ID#06** | **yes** | 2 | 7 | 3 | 4 | 0 |
| **ID#39** | **yes** | 13 | 16 | 9 | 14 | 8 |
| **ID#17** | **yes** | 14 | 19 | 4 | 15 | 9 |
| **ID#19** | **yes** | 6 | 4 | 8 | 15 | 8 |
| **ID#20** | **yes** | 2 |  | 12 | 4 | 2 |
| **ID#37** | **yes** | 26 | 5 | 0 | 16 | 15 |
| **ID#04** | **yes** | 24 | 8 | 10 | 34 | 35 |
| **ID#41** | **yes** | 13 | 15 | 20 | 25 | 15 |
| **ID#30** | **yes** | 6 | 14 | 14 | 12 | 12 |
| **ID#02** | **yes** | 31 | 31 | 36 | 26 | 30 |
| **ID#25** | **yes** | 0 | 1 |  | 4 | 8 |
| **ID#12** | **yes** | 11 | 16 | 10 | 22 | 10 |
| **ID#01** | **yes** | 20 | 20 | 16 | 17 | 16 |
| **ID#22** | **yes** | 14 | 12 | 8 | 18 | 15 |
| **ID#16** | **yes** | 8 | 10 | 6 | 8 | 4 |
| **ID#14** | **no** | 32 |  | 21 | 24 | 36 |
| **ID#36** | **no** | 5 | 19 | 13 | 13 | 20 |
| **ID#07** | **no** | 26 | 30 | 32 |  | 26 |
| **ID#31** | **no** | 16 | 30 | 33 | 20 | 10 |
| **ID#29** | **no** | 24 | 11 | 14 | 22 | 25 |
| **ID#11** | **no** | 27 | 25 | 14 | 27 | 34 |
| **ID#13** | **no** | 18 | 30 | 34 |  | 22 |
