# Supplementary material for: Effectiveness and implementation of an inpatient mental health care pathway at an epilepsy center: A prospective service evaluation
Source: Epilepsia. 2025 Nov 14;67(3):1358–70. doi: 10.1111/epi.70014 (PMC13007824; doi:10.1111/epi.70014)
Supplement: Supplementary file 3 — Table S6. [file EPI-67-1358-s001.docx]

**Supplementary Table 6**

|  | *RCI_1_*- | *RCI_1_*0 | *RCI_1_*+ | *RCI_3_*- | *RCI_3_*0 | *RCI_3_*+ | *RCI_6_*- | *RCI_6_*0 | *RCI_6_*+ | *RCI_12_*- | *RCI_12_*0 | *RCI_12_*+ |
| --- | --- | --- | --- | --- | --- | --- | --- | --- | --- | --- | --- | --- |
| Total Score | 6  (19%) | 17  (55%) | 8  (26%) | 1  (3%) | 15 (54%) | 12 (43%) | 4 (14%) | 15 (56%) | 8 (30%) | 3 (13%) | 14 (58%) | 7 (29%) |
| Seizure Worry | 1  (3%) | 27 (87%) | 3 (10%) | 1  (3%) | 24 (86%) | 3 (11%) | 0 | 25 (93%) | 2  (7%) | 1  (4%) | 20 (83%) | 3 (13%) |
| Overall QoL | 4 (13%) | 25 (81%) | 2  (7%) | 5 (18%) | 19 (68%) | 4 (14%) | 4 (15%) | 17 (63%) | 6 (22%) | 6 (25%) | 12 (50%) | 6 (25%) |
| Emotional Well-being | 2  (7%) | 22 (71%) | 7 (23%) | 1  (4%) | 21 (75%) | 6 (21%) | 0 | 24 (89%) | 3 (11%) | 2  (8%) | 15 (63%) | 7 (29%) |
| Energy/Fatigue | 2  (7%) | 21 (68%) | 8 (26%) | 2  (7%) | 22 (79%) | 4 (14%) | 2  (7%) | 20 (74%) | 5 (19%) | 1  (4%) | 19 (79%) | 4 (17%) |
| Cognition | 1  (3%) | 28 (90%) | 2  (7%) | 2  (7%) | 21 (75%) | 5 (18%) | 4 (15%) | 18 (67%) | 5 (19%) | 2  (8%) | 20 (83%) | 2  (8%) |
| Medication Effects | 2  (7%) | 26 (84%) | 3 (10%) | 3 (11%) | 19 (68%) | 6 (21%) | 3 (11%) | 21 (78%) | 3 (11%) | 2  (8%) | 17 (71%) | 5 (21%) |
| Social Function | 2  (7%) | 27 (87%) | 2  (7%) | 1  (3%) | 24 (86%) | 3 (11%) | 2  (7%) | 22 (82%) | 3 (11%) | 0 | 22 (92%) | 2  (8%) |

Reliable change indices after one (*RCI*_1_, N=31), three (*RCI*_3_, N=28), six (*RCI*_6_, N=27) and 12 months (*RCI*_12_, N=24).

*RCI-* = reliable deterioration, *RCI*0 = no reliable change, *RCI*+ = reliable improvement.

**Supplementary Table 7**

|  | *RCI_1_*- | *RCI_1_*0 | *RCI_1_*+ | *RCI_3_*- | *RCI_3_*0 | *RCI_3_*+ | *RCI_6_*- | *RCI_6_*0 | *RCI_6_*+ | *RCI_12_*- | *RCI_12_*0 | *RCI_12_*+ |
| --- | --- | --- | --- | --- | --- | --- | --- | --- | --- | --- | --- | --- |
| WSAS | 5 (16%) | 20 (65%) | 6 (19%) | 3 (11%) | 19 (70%) | 5 (19%) | 3 (11%) | 19 (70%) | 5 (19%) | 2 (9%) | 19 (86%) | 1 (5%) |

Reliable change indices after one (*RCI*_1_, N=31), three (*RCI*_3_, N=28), six (*RCI*_6_, N=27) and 12 months (*RCI*_12_, N=24).

*RCI-* = reliable deterioration, *RCI*0 = no reliable change, *RCI*+ = reliable improvement.
