# Supplementary material for: Effectiveness and implementation of an inpatient mental health care pathway at an epilepsy center: A prospective service evaluation
Source: Epilepsia. 2025 Nov 14;67(3):1358–70. doi: 10.1111/epi.70014 (PMC13007824; doi:10.1111/epi.70014)
Supplement: Supplementary file 4 — Table S8. [file EPI-67-1358-s002.docx]

**Supplementary Table 8**

|  | *RCI_1_*- | *RCI_1_*0 | *RCI_1_*+ | *RCI_3_*- | *RCI_3_*0 | *RCI_3_*+ | *RCI_6_*- | *RCI_6_*0 | *RCI_6_*+ | *RCI_12_*- | *RCI_12_*0 | *RCI_12_*+ |
| --- | --- | --- | --- | --- | --- | --- | --- | --- | --- | --- | --- | --- |
| BDI | 2 (7%) | 25 (80%) | 4 (13%) | 0 | 24 (86%) | 4 (14%) | 0 | 23 (85%) | 4 (15%) | 2 (8%) | 18 (75%) | 4 (17%) |
| BAI | 3 (10%) | 22 (71%) | 6 (19%) | 0 | 21 (75%) | 7 (25%) | 3 (11%) | 19 (70%) | 5 (19%) | 3 (12%) | 17 (71%) | 4 (17%) |

Reliable change indices after one (*RCI*_1_, N=31), three (*RCI*_3_, N=28), six (*RCI*_6_, N=27) and 12 months (*RCI*_12_, N=24).

BDI = Beck Depression Inventory II, BAI = Beck Anxiety Inventory, *RCI-* = reliable deterioration, *RCI*0 = no reliable change, *RCI*+ = reliable improvement.
