# Supplementary material for: Effectiveness and implementation of an inpatient mental health care pathway at an epilepsy center: A prospective service evaluation
Source: Epilepsia. 2025 Nov 14;67(3):1358–70. doi: 10.1111/epi.70014 (PMC13007824; doi:10.1111/epi.70014)
Supplement: Supplementary file 5 — Table S9. [file EPI-67-1358-s003.docx]

|  | **Supplementary Table 9** | | | | | |
| --- | --- | --- | --- | --- | --- | --- |
|  | **BDI** | **BAI** | **QOLIE-31** | **WSAS** | **adherence** | **sz frequency** |
| **1** | ↑ | ↔ | ↑ | ↔ | yes | ↔ |
| **2** | ↔ | ↓ | ↔ | ↔ | yes | ↔ |
| **3** | ↔ | ↔ | ↔ | ↔ | yes | ↔ |
| **4** | ↔ | ↔ | ↓ | ↔ | yes | ↑ |
| **5** | ↔ | ↔ | ↔ | ↔ | yes | ↔ |
| **6** | ↔ | ↑ | ↑ | ↔ | yes | ↑ |
| **7** | ↔ | ↔ | ↔ | ↔ | no | ↔ |
| **8** | ↔ | ↔ | ↔ | ↑ | yes | ↑ |
| **9** | ↔ | ↔ | ↔ | ↔ | no | ↑ |
| **10** | ↔ | ↔ | ↑ | ↔ | yes | ↑ |
| **11** | ↑ | ↑ | ↔ | ↔ | yes | ↓ |
| **12** | ↑ | ↑ | ↑ | ↔ | yes | ↑ |
| **13** | ↔ | ↑ | ↔ | ↓ | yes | ↔ |
| **14** | ↔ | ↔ | ↔ | ↔ | no | ↔ |
| **15** | ↑ | ↔ | ↔ | ↔ | yes | ↑ |
| **16** | ↓ | ↓ | ↓ | ↓ | no | ↔ |
| **17** | ↔ | ↔ | ↑ | ↔ | yes | no |
| **18** | ↔ | ↓ | ↔ | ↔ | yes | ↑ |
| **19** | ↔ | ↔ | ↔ | ↔ | yes | ↑ |
| **20** | ↔ | ↔ | ↓ | ↔ | no | ↔ |
| **21** | ↓ | ↔ | ↔ | ↔ | no | ↑ |
| **22** | ↔ | ↔ | ↑ | ↔ | yes | ↔ |
| **23** | ↔ | ↔ | ↔ | ↔ | no | ↔ |
| **24** | ↔ | ↔ | ↑ | ↔ | yes | ↑ |

↑: improvement, ↔: no change, ↓: worsening, sz: seizure, BDI: Becks Depression Inventory, BAI: Becks Anxiety Inventory, QOLIE-31: Quality of life in epilepsy, WSAS: Work and social adjustment scale
